# Baseline BMI and weight change in dilated cardiomyopathy: associations with LVEF, BNP, and NYHA class

**DOI:** 10.3389/fcvm.2026.1901876

**Published:** 2026-09-25

**Authors:** Xia Sun, Xiang Tang, Mingfeng Jin, Wei Yuan, Guoyue Yuan

**Affiliations:** 1 Department of Cardiology, Affiliated Hospital of Jiangsu University, Zhenjiang, Jiangsu, China; 2 Department of Endocrinology and Metabolism, Affiliated Hospital of Jiangsu University, Zhenjiang, Jiangsu, China

**Keywords:** body mass index, dilated cardiomyopathy, heart failure, obesity, reverse remodeling, weight change

## Abstract

**Background and Aims:**

Baseline body mass index (BMI) and longitudinal weight change capture different clinical dimensions. We examined their concurrent associations with 12-month changes in left ventricular ejection fraction (LVEF), B-type natriuretic peptide (BNP), and New York Heart Association (NYHA) functional class among patients with dilated cardiomyopathy.

**Methods:**

Of 600 records screened, 322 patients had complete paired baseline and 12-month measurements and were included in this retrospective cohort analysis. Percentage weight loss was calculated as 100 × (baseline weight−12-month weight)/baseline weight; negative values denoted weight gain. Joint models included baseline BMI and weight loss and adjusted for age, sex, log-transformed baseline BNP, baseline LVEF, left ventricular end-diastolic diameter, baseline NYHA class, diabetes, chronic kidney disease, atrial fibrillation, and coronary atherosclerosis.

**Results:**

Thirty-six patients gained weight, six demonstrated no weight change, and 280 lost weight. Baseline BMI was not independently associated with LVEF change (*β* = −0.099, 95% CI −0.269 to 0.071; *P* = 0.254), BNP log ratio (*β* = 0.008, 95% CI −0.014 to 0.030; *P* = 0.467), or NYHA improvement (OR = 1.02, 95% CI 0.94–1.10; *P* = 0.709). Each 5% greater weight loss was concurrently associated with a greater increase in LVEF (*β* = 2.43 percentage points, 95% CI 1.66–3.20), a lower BNP log ratio (*β* = −0.317, 95% CI −0.415 to −0.219), and higher odds of NYHA improvement (OR = 1.93, 95% CI 1.38–2.74; all *P* < 0.001). The associations remained consistent after adjustment for the availability of heart-failure and weight-management therapies.

**Conclusions:**

Twelve-month weight change was associated with concurrent changes in LVEF, BNP, and functional status, whereas baseline BMI was not. These data do not establish temporal direction, causality, or the superiority of weight change as a clinical marker.

## Graphical Abstract

**Figure:**
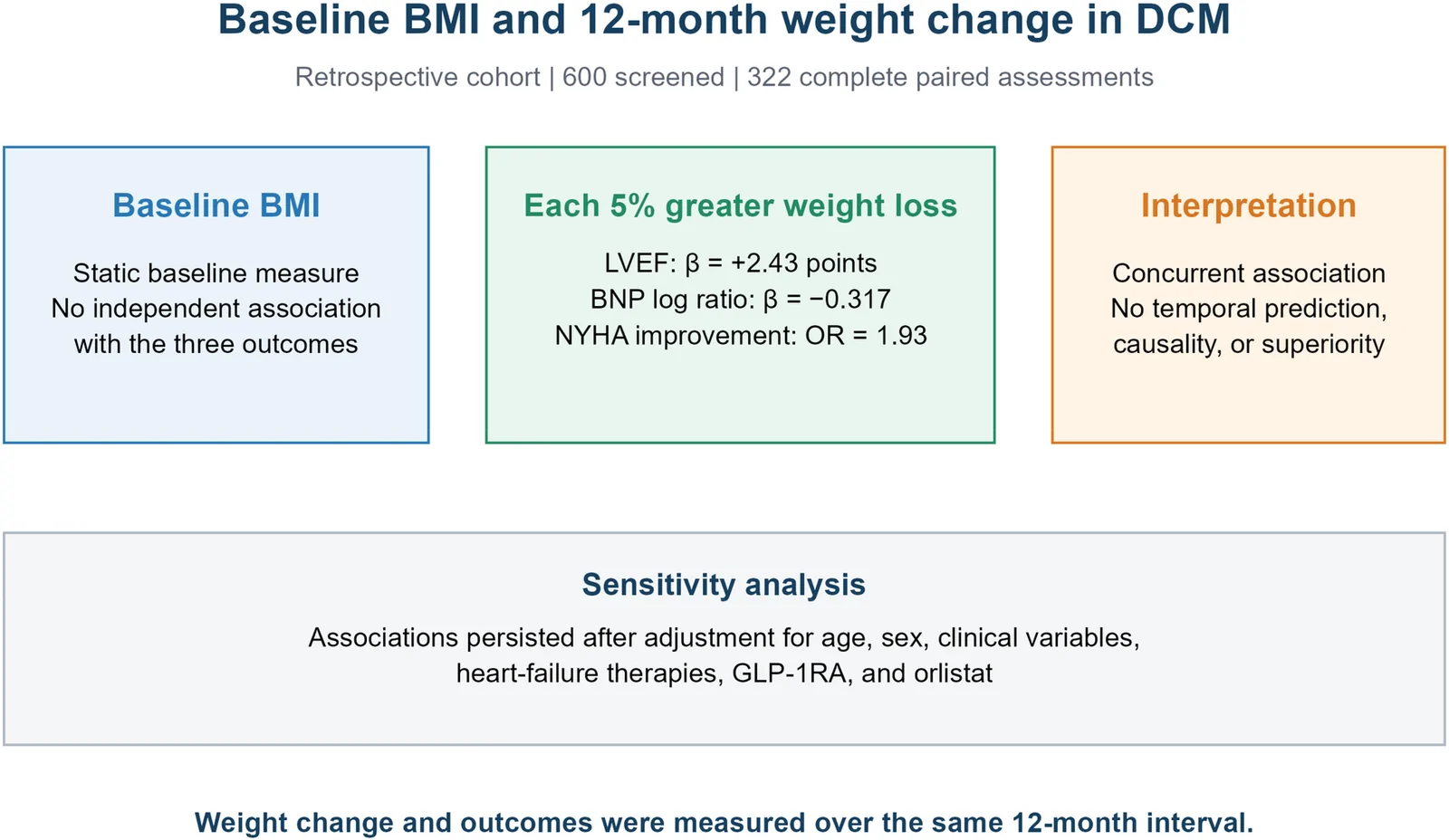

## Introduction

Dilated cardiomyopathy (DCM) is a heterogeneous myocardial disorder in which prognosis is influenced by myocardial substrate, inflammatory activity, arrhythmia burden, imaging phenotype, and response to contemporary heart-failure therapy (1–10). Changes in left ventricular ejection fraction (LVEF), natriuretic peptides, and functional status are clinically informative markers of disease trajectory, but they do not constitute a complete structural reverse-remodeling phenotype.

Obesity is common in cardiovascular disease and heart failure, yet body mass index (BMI) cannot distinguish adipose distribution, lean mass, edema, sarcopenia, nutritional reserve, or cachexia (11–14). Consequently, a single baseline BMI measurement may not adequately capture subsequent changes in cardiometabolic status or intravascular volume.

Longitudinal weight change may provide complementary information, but it is not etiologically specific. Weight loss can reflect intentional fat loss, decongestion, treatment effects, clinical recovery, progressive disease, or catabolic illness. Studies in obesity-related heart-failure phenotypes have associated weight reduction with changes in symptoms, natriuretic peptides, and cardiac structure (15–18), and decision-analytic work has explored its potential implications for advanced heart-failure therapy (19). Incretin-based therapies may also affect cardiovascular pathways beyond weight reduction alone (20). However, these observations do not establish a treatment effect in DCM.

We therefore evaluated baseline BMI and 12-month weight change in the same set of adjusted models to assess concurrent changes in LVEF, B-type natriuretic peptide (BNP), and New York Heart Association (NYHA) functional class. Because weight change and outcomes were measured over the same interval, the analysis was explicitly framed as associational rather than predictive or causal.

## Materials and methods

### Study design and population

This single-center retrospective observational study analyzed 600 patient records from those who received routine care between January 2024 and May 2025. At eligibility assessment, 245 records were excluded for the following mutually exclusive primary reasons: DCM was not confirmed or another cardiomyopathy was present (*n* = 150); ischemic heart disease was sufficient to explain left ventricular dysfunction (*n* = 40); recent chemotherapy or immunosuppressive therapy, or severe infection, was documented (*n* = 40); or a weight-affecting disorder or medication, pregnancy, or lactation was present (*n* = 15). The remaining 355 patients entered a 12-month follow-up. During follow-up, 13 patients were excluded because paired 12-month measurements were incomplete or they were lost to follow-up, and 20 were excluded after treatment with (cardiac resynchronization therapy with or without defibrillator) CRT/CRT-D, cardiac contractility modulation, or a left ventricular assist device. Thus, 278 of the 600 screened records were excluded before analysis. The final complete-case cohort comprised 322 patients with paired baseline and 12-month measurements of weight, LVEF, BNP, and NYHA class (Figure 1). Follow-up completeness was verified for every included patient, and the database was locked on 31 May 2026. Individual-level baseline data were not available for excluded records; therefore, included and excluded patients could not be compared.

**Figure 1:**
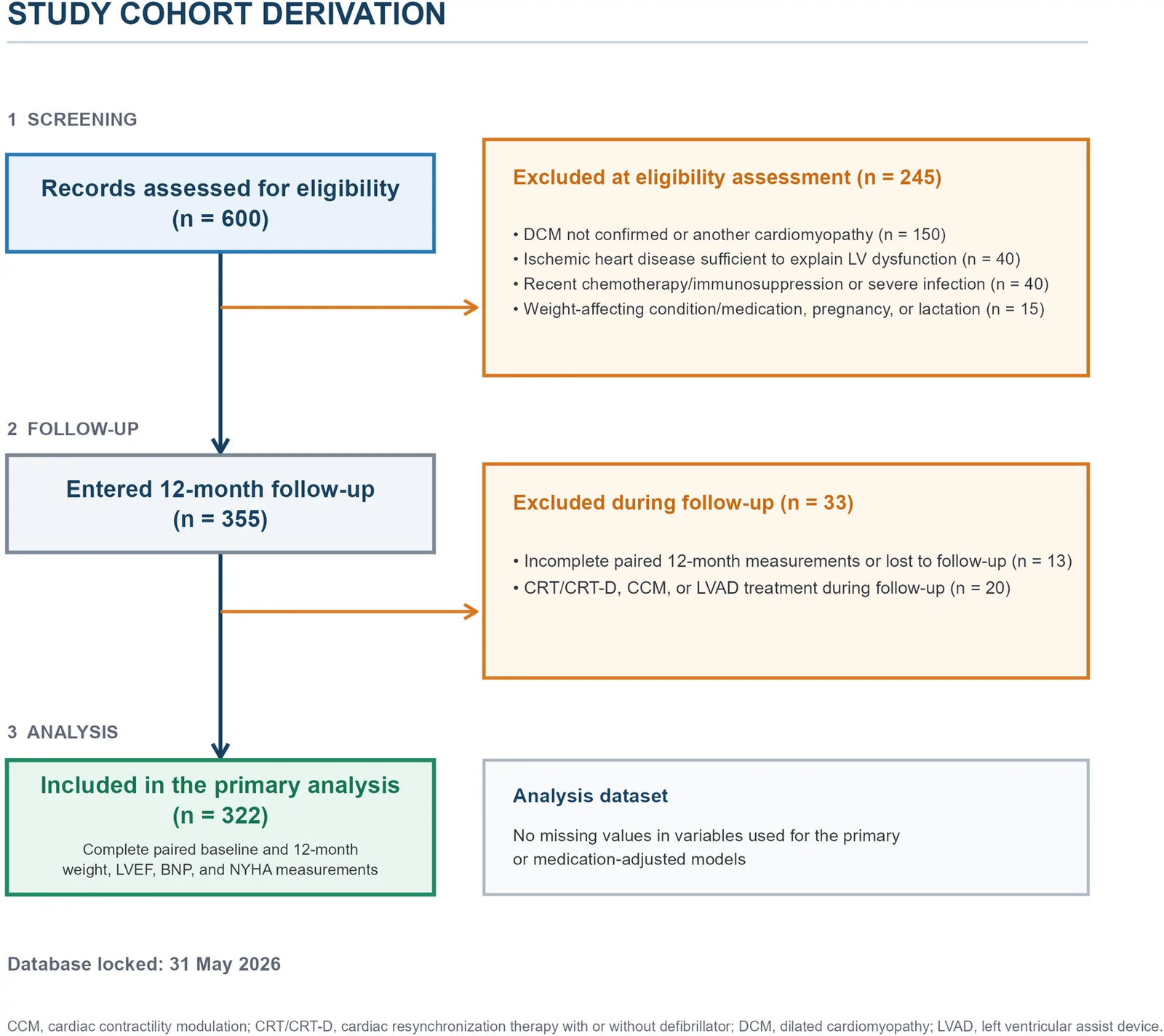
Study cohort derivation. Eligibility exclusions and exclusions during follow-up are presented separately. Each excluded patient was assigned one mutually exclusive primary reason. CCM, cardiac contractility modulation; CRT/CRT-D, cardiac resynchronization therapy with or without defibrillator; DCM, dilated cardiomyopathy; LVAD, left ventricular assist device.

### Eligibility and DCM ascertainment

Eligible patients were adults older than 18 years with a clinical diagnosis of DCM, NYHA functional class I–IV, guideline-directed cardiomyopathy therapy as clinically appropriate, complete clinical records, and adequate cognitive and clinical capacity for longitudinal follow-up (21–23). The treating cardiology team diagnosed DCM based on left ventricular dilatation and systolic dysfunction not explained by abnormal loading conditions or coronary artery disease sufficient to cause an ischemic cardiomyopathy (1). Exclusion criteria included another cardiomyopathy or ischemic heart disease sufficient to explain the phenotype; immunosuppressive or chemotherapy treatment in the preceding 6 months; eating disorders; endocrine conditions or medications materially affecting weight, including thyroid dysfunction and systemic glucocorticoids; pregnancy or lactation; severe infection; or clinical deterioration during follow-up requiring CRT/CRT-D, cardiac contractility modulation, or left ventricular assist-device treatment. Coronary atherosclerosis was retained as a comorbidity only when it was not considered the cause of the DCM phenotype. Overweight and obesity were classified using Chinese adult BMI thresholds; these categories were not universal eligibility requirements because the analysis included the full BMI spectrum. Quantitative indexed ventricular volume thresholds, detailed coronary stenosis thresholds, cardiac magnetic resonance imaging, genetic testing, myocarditis work-up, and centralized etiological adjudication were not consistently available.

### Anthropometric measurements and outcomes

Baseline weight was recorded during the index admission, and follow-up weight was recorded at the scheduled 12-month assessment. BMI was calculated as weight in kilograms divided by height in meters squared. Percentage weight loss was calculated as 100 × (baseline weight−12-month weight)/baseline weight; positive values indicated weight loss, while negative values indicated weight gain. Weight gain was retained as a negative continuous value. In threshold analyses, weight gain and no weight change were included in the <5% reference category. The outcomes were a change in LVEF, the natural logarithm of the follow-up-to-baseline BNP ratio, and an improvement in NYHA class. These outcomes were interpreted as markers related to reverse remodeling rather than reverse remodeling itself (24). The aggregate major adverse cardiovascular events field was not analyzed because component identities and event dates were unavailable to establish temporal ordering relative to the 12-month measurements.

### Echocardiography

Transthoracic echocardiography was performed at baseline and 12 months under a standardized departmental acquisition and reporting protocol. LVEF was calculated using the biplane method of disks (modified Simpson method) from apical four- and two-chamber views, with endocardial borders traced at end-diastole and end-systole in accordance with ASE/EACVI recommendations (25). All LVEF measurements were independently performed by two physicians in the Department of Echocardiography, both at the rank of associate chief physician. The studies were anonymized, and each reader was masked to the patient's clinical and laboratory data, treatment information, and the other reader's measurement. The final LVEF documented in the echocardiography report was used for analysis. Separate reader-level measurements were not retained in the analysis dataset; therefore, a formal interobserver intraclass correlation coefficient could not be calculated retrospectively.

### Medication exposure

Available indicators of ACEI/ARB/ARNI, β-receptor blocker, sodium-glucose cotransporter-2 inhibitor, mineralocorticoid receptor antagonist, and soluble guanylate cyclase stimulator use during follow-up were included in the treatment-adjusted sensitivity analysis. When clinically indicated, GLP-1 receptor agonists or orlistat were initiated after admission following joint assessment by the cardiology and endocrinology teams and were continued for 12 months. Serial dose changes, loop-diuretic dose, and objective longitudinal congestion measurements were unavailable.

### Statistical analysis

Continuous variables are summarized as mean ± standard deviation or median [interquartile range], and categorical variables are summarized as *n* (%). Across baseline BMI categories, variables summarized as mean ± standard deviation were compared using one-way analysis of variance, variables summarized as medians were compared using the Kruskal–Wallis test, and categorical variables were compared using the chi-square or Fisher's exact test, as appropriate. Baseline BMI and 12-month weight loss were entered together in all primary models. Models adjusted for age, sex, natural-log baseline BNP, baseline LVEF, left ventricular end-diastolic diameter, baseline NYHA class, diabetes, chronic kidney disease, atrial fibrillation, and coronary atherosclerosis. Linear regression was used to model LVEF change and BNP log ratio, and logistic regression was used to model NYHA improvement. A sensitivity analysis additionally adjusted for the use of available heart-failure therapies, GLP-1 receptor agonists, and orlistat. Threshold analyses compared 5%–9.9% and ≥10% weight loss with <5% weight loss, as well as weight gain and no change. Collinearity was assessed using the Pearson correlation coefficient and variance inflation factors. All tests were two-sided, with *P* < 0.05 considered statistically significant. Analyses were exploratory, and no adjustment for multiple testing was applied. Analyses were performed using R version 4.5.1.

## Results

### Cohort characteristics

Of 600 screened records, 322 were included and 278 were excluded before analysis (Figure 1). The included cohort had no missing values in the variables used in the primary or medication-adjusted models. The mean age was 60.0 ± 13.1 years, and 220 (68.3%) patients were men. At baseline, 84 patients had a BMI <24 kg/m^2^, 132 had a BMI of 24–27.9 kg/m^2^, and 106 had a BMI ≥28 kg/m^2^. Median weight loss was 5.6% [3.3%–8.0%]. Thirty-six patients gained weight, six showed no weight change, and 280 lost weight. The <5%, 5%–9.9%, and ≥10% weight-loss categories included 122, 150, and 50 patients, respectively (Table 1).

**Table 1:** Characteristics of the included cohort by baseline BMI category.

Variable	Overall (*n* = 322)	BMI <24 (*n* = 84)	BMI 24–27.9 (*n* = 132)	BMI ≥28 (*n* = 106)	*P*-value
Age (years)	60.0 ± 13.1	67.0 ± 8.3	59.0 ± 12.6	55.6 ± 14.7	<0.001
Male sex, *n* (%)	220 (68.3)	54 (64.3)	95 (72.0)	71 (67.0)	0.465
BMI (kg/m^2^)	26.6 [23.9, 28.4]	21.5 [20.3, 22.9]	26.3 [25.2, 27.1]	29.7 [28.5, 31.6]	<0.001
12-month weight loss (%)	5.6 [3.3, 8.0]	3.4 [1.1, 6.3]	6.2 [4.1, 8.6]	6.5 [4.6, 8.8]	<0.001
Baseline LVEF (%)	32.4 ± 8.6	32.6 ± 10.9	32.0 ± 8.3	32.6 ± 7.0	0.849
LVEF change, percentage points	6.6 ± 6.7	6.4 ± 7.1	7.0 ± 6.4	6.2 ± 6.8	0.655
Baseline BNP (pg/mL)	973.0 [469.0, 2,039.0]	1,055.0 [321.0, 2,902.5]	936.5 [533.0, 1,970.0]	992.5 [566.0, 1,980.0]	0.933
BNP log ratio, follow-up/baseline	−0.9 ± 0.8	−0.7 ± 0.7	−1.0 ± 0.9	−0.8 ± 0.8	0.022
NYHA class improved, *n* (%)	231 (71.7)	54 (64.3)	96 (72.7)	81 (76.4)	0.173
Diabetes, *n* (%)	91 (28.3)	13 (15.5)	41 (31.1)	37 (34.9)	0.008
Chronic kidney disease, *n* (%)	23 (7.1)	9 (10.7)	8 (6.1)	6 (5.7)	0.333
Atrial fibrillation, *n* (%)	77 (23.9)	15 (17.9)	35 (26.5)	27 (25.5)	0.313
Coronary atherosclerosis, *n* (%)	58 (18.0)	22 (26.2)	23 (17.4)	13 (12.3)	0.045
ACEI/ARB/ARNI during follow-up, *n* (%)	305 (94.7)	78 (92.9)	127 (96.2)	100 (94.3)	0.510
β-receptor blockers during follow-up, *n* (%)	315 (97.8)	82 (97.6)	129 (97.7)	104 (98.1)	1.000
SGLT2 inhibitors during follow-up, *n* (%)	311 (96.6)	80 (95.2)	129 (97.7)	102 (96.2)	0.588
Mineralocorticoid receptor antagonists, *n* (%)	314 (97.5)	83 (98.8)	130 (98.5)	101 (95.3)	0.278
Soluble guanylate cyclase stimulators, *n* (%)	307 (95.3)	81 (96.4)	130 (98.5)	96 (90.6)	0.015
GLP-1 receptor agonists for 12 months, *n* (%)	113 (35.1)	5 (6.0)	28 (21.2)	80 (75.5)	<0.001
Orlistat for 12 months, *n* (%)	64 (19.9)	4 (4.8)	26 (19.7)	34 (32.1)	<0.001

Values are mean ± SD, median [IQR], or *n* (%). *P*-values are descriptive. Medication indicators denote available use during follow-up; GLP-1RA and orlistat were initiated after admission when clinically indicated and continued for 12 months.

### Joint associations of BMI and weight change

In joint adjusted models, baseline BMI was not independently associated with LVEF change (*β* = −0.099, 95% CI −0.269 to 0.071; *P* = 0.254), BNP log ratio (*β* = 0.008, 95% CI −0.014 to 0.030; *P* = 0.467), or NYHA improvement (OR = 1.02, 95% CI 0.94–1.10; *P* = 0.709). Each 5% greater weight loss was concurrently associated with a greater increase in LVEF (*β* = 2.43 percentage points, 95% CI 1.66–3.20), a lower BNP log ratio (*β* = −0.317, 95% CI −0.415 to −0.219), and higher odds of NYHA improvement (OR = 1.93, 95% CI 1.38–2.74; all *P* < 0.001; Table 2 and Figures 2, 3).

**Table 2:** Joint adjusted associations of baseline BMI and 12-month weight loss.

Outcome	Exposure	Adjusted estimate (95% CI)	*P*-value	*N*
LVEF change	Baseline BMI, per 1 kg/m^2^	*β* = −0.099 (−0.269 to 0.071)	0.254	322
LVEF change	Weight loss, per 5%	*β* = 2.428 (1.657 to 3.200)	<0.001	322
BNP log ratio	Baseline BMI, per 1 kg/m^2^	*β* = 0.008 (−0.014 to 0.030)	0.467	322
BNP log ratio	Weight loss, per 5%	*β* = −0.317 (−0.415 to −0.219)	<0.001	322
NYHA improvement	Baseline BMI, per 1 kg/m^2^	OR = 1.02 (0.94 to 1.10)	0.709	322
NYHA improvement	Weight loss, per 5%	OR = 1.93 (1.38 to 2.74)	<0.001	322

Models include both exposures and adjust for age, sex, log baseline BNP, baseline LVEF, LVEDD, baseline NYHA class, diabetes, chronic kidney disease, atrial fibrillation, and coronary atherosclerosis. *β* denotes the adjusted regression coefficient; OR denotes the adjusted odds ratio.

LVEDD, left ventricular end-diastolic dimension.

**Figure 2:**
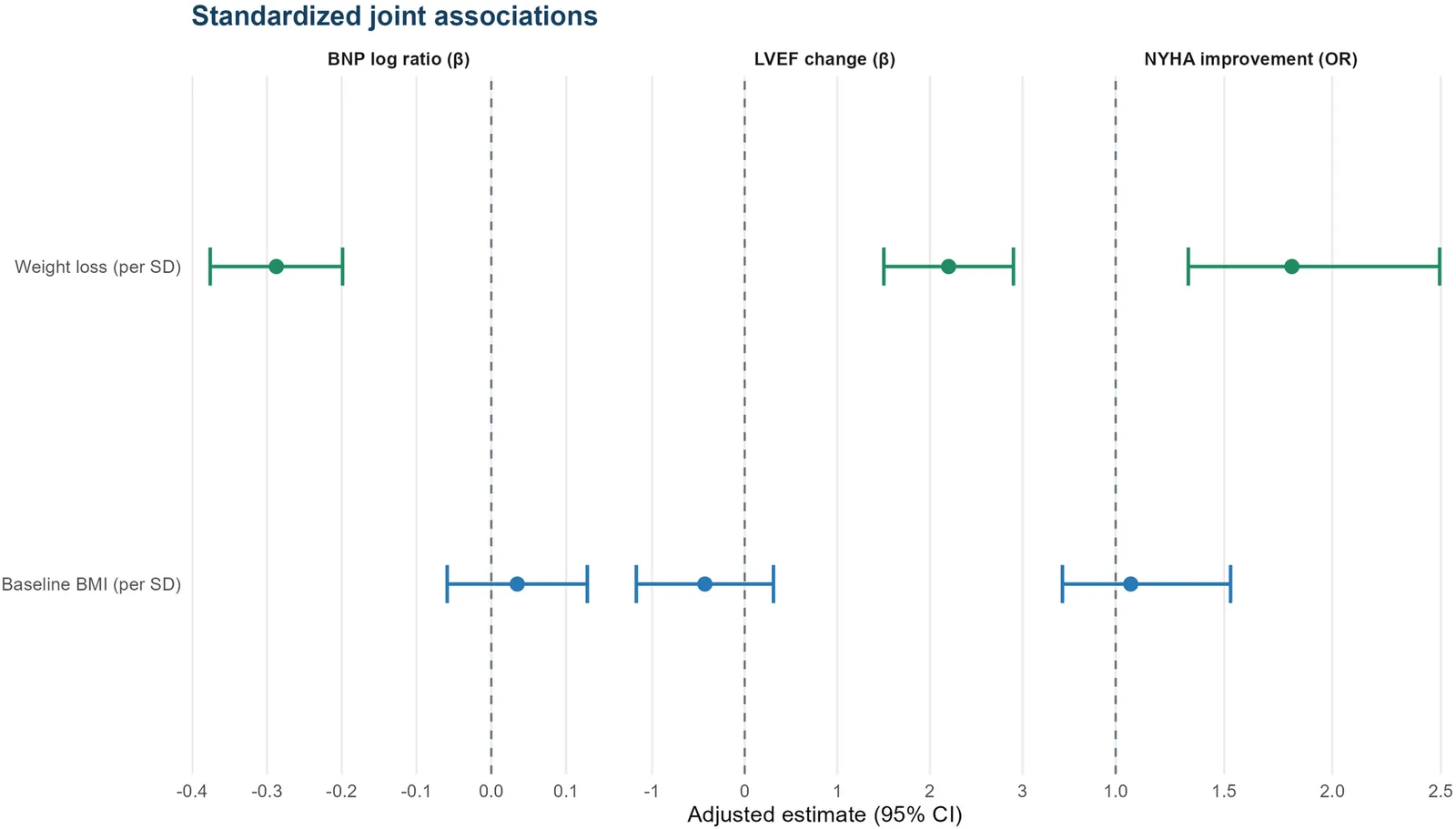
Standardized joint associations of baseline BMI and 12-month weight loss with concurrent changes in LVEF, BNP, and NYHA class. Points are adjusted estimates, and bars are 95% confidence intervals.

**Figure 3:**
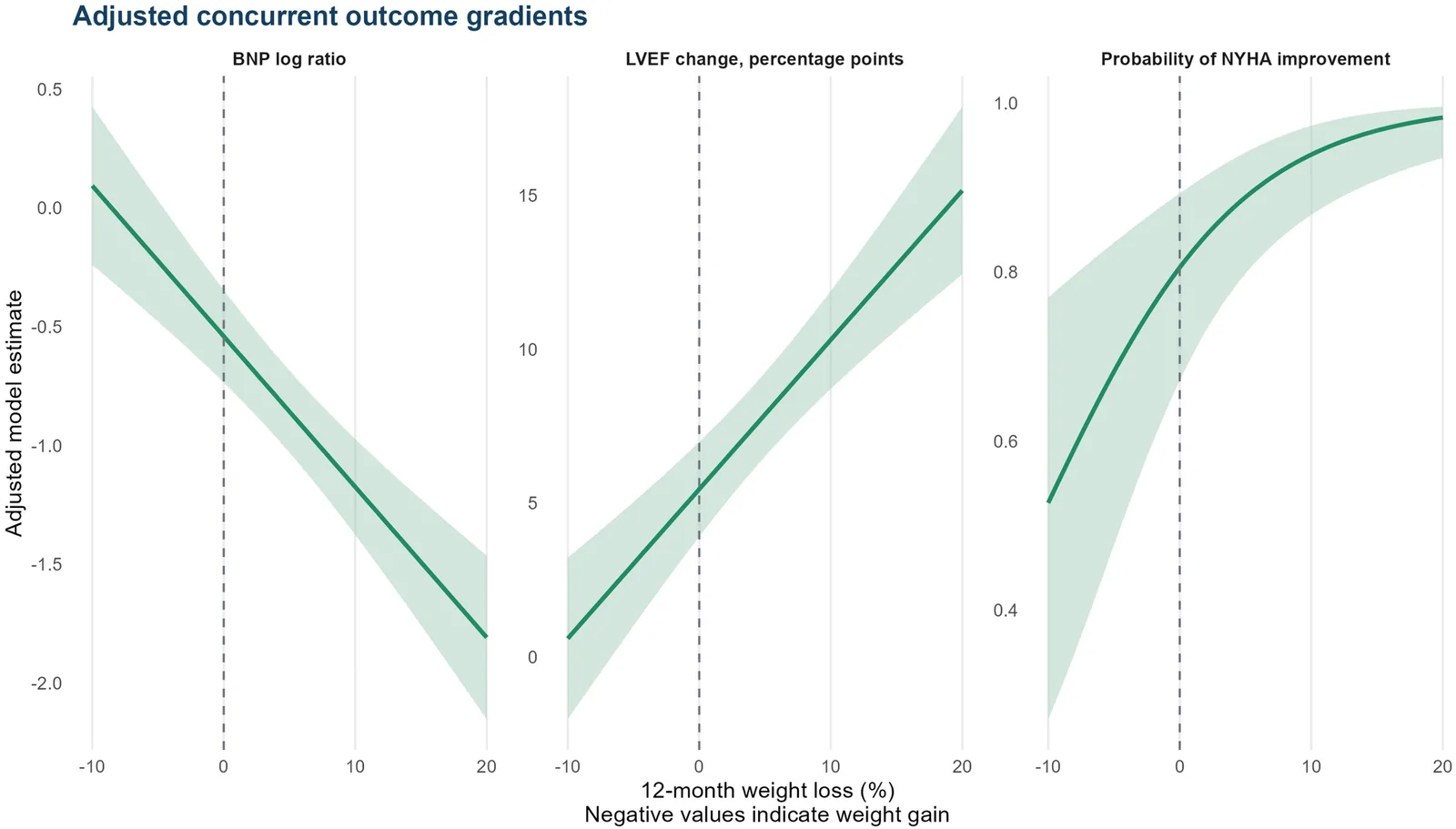
Adjusted concurrent outcome gradients across 12-month weight loss. Negative values indicate weight gain. Shading denotes 95% confidence intervals.

### Sensitivity analyses and collinearity

After further adjustment for the use of available heart-failure therapies, GLP-1 receptor agonists, and orlistat, each 5% greater weight loss remained associated with LVEF change (*β* = 2.26, 95% CI 1.48–3.03), BNP log ratio (*β* = −0.295, 95% CI −0.394 to −0.197), and NYHA improvement (OR = 1.85, 95% CI 1.31–2.65; all *P* < 0.001), whereas BMI remained unassociated with each outcome (Table 3). Threshold analyses showed graded associations across weight-loss categories (Supplementary Table S1). The correlation between BMI and weight loss was 0.275; variance inflation factors were 1.28 for BMI and 1.15 for weight loss.

**Table 3:** Sensitivity analysis additionally adjusted for available therapies.

Outcome	Exposure	Adjusted estimate (95% CI)	*P*-value	*n*
LVEF change	Baseline BMI, per 1 kg/m^2^	*β* = −0.114 (−0.307 to 0.079)	0.247	322
LVEF change	Weight loss, per 5%	*β* = 2.256 (1.482 to 3.030)	<0.001	322
BNP log ratio	Baseline BMI, per 1 kg/m^2^	*β* = 0.016 (−0.009 to 0.041)	0.201	322
BNP log ratio	Weight loss, per 5%	*β* = −0.295 (−0.394 to −0.197)	<0.001	322
NYHA improvement	Baseline BMI, per 1 kg/m^2^	OR = 1.01 (0.92 to 1.11)	0.857	322
NYHA improvement	Weight loss, per 5%	OR = 1.85 (1.31 to 2.65)	<0.001	322

Models additionally adjust for the use of ACEI/ARB/ARNI, β-receptor blockers, SGLT2 inhibitors, mineralocorticoid receptor antagonists, soluble guanylate cyclase stimulators, GLP-1RA, and orlistat. *β* denotes the adjusted regression coefficient; OR denotes the adjusted odds ratio.

## Discussion

In this complete-case DCM cohort, 12-month weight change, but not baseline BMI, was associated with concurrent changes in LVEF, BNP, and NYHA class after adjustment for age, sex, baseline disease markers, comorbidities, and available treatment indicators. Baseline BMI is a static measure, whereas weight change accumulates over the follow-up period; the present findings therefore do not establish that weight change is a superior baseline marker.

The observed associations are clinically plausible but etiologically ambiguous. Weight loss may reflect intentional weight management, decongestion, medication effects, improved clinical status, reduced appetite, progressive disease, or catabolic illness. Conversely, clinical improvement and decongestion may themselves lead to weight loss. Because anthropometric and outcome changes were measured across the same 12-month interval, their temporal order and causal direction cannot be determined.

Trials in obesity-related heart failure with preserved ejection fraction have associated incretin-based weight reduction with changes in symptoms, natriuretic peptides, and cardiac structure (15–18), and Rouhi et al. modeled the potential effects of metabolic and bariatric surgery on advanced heart-failure outcomes (19). Those populations and interventions differ from DCM, and our observational analysis does not identify weight loss as a therapeutic target. In clinical practice, weight trajectories should be interpreted alongside congestion, nutritional status, body composition, medication exposure, and overall disease course.

The analysis also underscores the importance of correctly defining longitudinal exposures. The signed formula retained 36 instances of weight gain as negative values; an unsigned magnitude would have misclassified weight gain as weight loss. The consistency of the associations after adjustment for available therapies and across categorical thresholds supports the stability of the statistical pattern, although residual confounding remains likely.

### Strengths and limitations

Strengths of this study include verification of the signed weight-change exposure, joint modeling of baseline BMI and weight change, complete paired measurements in the analyzed cohort, and sensitivity analyses incorporating available treatment indicators. However, several limitations remain. First, this was a single-center retrospective complete-case analysis. Although 600 records were screened and 278 were excluded for the reasons listed in Figure 1, individual-level baseline data for excluded patients were unavailable, precluding a formal included-versus-excluded comparison and leaving selection and survivor bias possible. Second, exposure and outcomes were measured over the same interval. Third, intentional fat loss, decongestion, cachexia, and other causes of weight change were not adjudicated; body composition, waist circumference, nutritional indices, edema, serial diuretic dosed, and objective measures of congestion were unavailable. Fourth, follow-up ventricular volumes or dimensions, global longitudinal strain, mitral regurgitation, cardiac magnetic resonance imaging, and genetic testing were unavailable; LVEF, BNP, and NYHA changes are therefore markers of reverse remodeling rather than a complete structural phenotype. Fifth, LVEF was independently assessed by two blinded readers, but separate reader-level measurements were not retained, and a formal interobserver intraclass correlation coefficient was not available retrospectively. Finally, medication indicators did not capture serial dose changes, and residual confounding and measurement variability remain possible.

## Conclusion

In this retrospective DCM cohort, 12-month weight change was associated with concurrent changes in LVEF, BNP, and NYHA functional class, whereas baseline BMI was not independently associated with these outcomes. These findings do not establish temporal prediction, causality, the clinical superiority of weight change, or a weight-loss treatment target. Prospective studies with repeated measurements of body composition, congestion, treatment, and structural cardiac are needed.

## Data Availability

The original contributions presented in the study are included in the article/Supplementary Material, further inquiries can be directed to the corresponding author.
