# Supplementary material for: Baseline BMI and weight change in dilated cardiomyopathy: associations with LVEF, BNP, and NYHA class
Source: Front Cardiovasc Med. 2026 Sep 25;13:1901876. doi: 10.3389/fcvm.2026.1901876 (PMC13649749; doi:10.3389/fcvm.2026.1901876)
Supplement: Supplementary file 1 [file Table1.pdf]

Supplementary Table S1. Weight-loss category sensitivity analysis

Outcome	Category	Adjusted estimate (95% CI)	P value	N
LVEF change	5–9.9% vs <5%	β = 2.336 (0.812 to 3.859)	0.003	322
LVEF change	≥10% vs <5%	β = 4.924 (2.797 to 7.051)	<0.001	322
BNP log ratio	5–9.9% vs <5%	β = −0.210 (−0.405 to −0.016)	0.034	322
BNP log ratio	≥10% vs <5%	β = −0.577 (−0.849 to −0.305)	<0.001	322
NYHA improvement	5–9.9% vs <5%	OR = 2.33 (1.21 to 4.56)	0.012	322
NYHA improvement	≥10% vs <5%	OR = 3.08 (1.20 to 8.49)	0.023	322
